# Effect of the Target and Conflicting Frequency and Time Ranges on Consonant Enhancement in Normal-Hearing Listeners

**DOI:** 10.3389/fpsyg.2021.733100

**Published:** 2021-11-15

**Authors:** Yang-Soo Yoon

**Affiliations:** Laboratory of Translational Auditory Research, Department of Communication Sciences and Disorders, Baylor University, Waco, TX, United States

**Keywords:** spectral cues, temporal cues, articulation-index gram, conflicting ranges, target ranges, consonant recognition

## Abstract

In this paper, the effects of intensifying useful frequency and time regions (target frequency and time ranges) and the removal of detrimental frequency and time regions (conflicting frequency and time ranges) for consonant enhancement were determined. Thirteen normal-hearing (NH) listeners participated in two experiments. In the first experiment, the target and conflicting frequency and time ranges for each consonant were identified under a quiet, dichotic listening condition by analyzing consonant confusion matrices. The target frequency range was defined as the frequency range that provided the highest performance and was decreased 40% from the peak performance from both high-pass filtering (HPF) and low-pass filtering (LPF) schemes. The conflicting frequency range was defined as the frequency range that yielded the peak errors of the most confused consonants and was 20% less than the peak error from both filtering schemes. The target time range was defined as a consonant segment that provided the highest performance and was decreased 40% from that peak performance when the duration of the consonant was systematically truncated from the onset. The conflicting time ranges were defined on the coincided target time range because, if they temporarily coincide, the conflicting frequency ranges would be the most detrimental factor affecting the target frequency ranges. In the second experiment, consonant recognition was binaurally measured in noise under three signal processing conditions: unprocessed, intensified target ranges by a 6-dB gain (target), and combined intensified target and removed conflicting ranges (target-conflicting). The results showed that consonant recognition improved significantly with the target condition but greatly deteriorated with a target-conflicting condition. The target condition helped transmit voicing and manner cues while the target-conflicting condition limited the transmission of these cues. Confusion analyses showed that the effect of the signal processing on consonant improvement was consonant-specific: the unprocessed condition was the best for /da, pa, ma, sa/; the target condition was the best for /ga, fa, va, za, ʒa/; and the target-conflicting condition was the best for /na, ʃa/. Perception of /ba, ta, ka/ was independent of the signal processing. The results suggest that enhancing the target ranges is an efficient way to improve consonant recognition while the removal of conflicting ranges negatively impacts consonant recognition.

## Introduction

Consonant recognition depends on the listener’s ability to discriminate details of spectral and temporal acoustic cues such as voicing, an onset of the noise burst, and spectral and temporal transitions (Miller and Nicely, 1955; Stevens and Klatt, 1974; Stevens and Blumstein, 1978; Blumstein and Stevens, 1979, 1980). Many classic studies used synthetic consonants, which require prior knowledge of the spectral and temporal cues that are critical for perception (Hughes and Halle, 1956; Heinz and Stevens, 1961; Blumstein et al., 1977; Stevens and Blumstein, 1978). Other studies used naturally produced consonants and identified the spectral and temporal cues for recognition (Soli, 1981; Baum and Blumstein, 1987; Behrens and Blumstein, 1988; Jongman et al., 2000). While the results obtained from these studies help characterize spectral and temporal cues and their variability, there are limited studies available that utilize these identified cues for an enhancement of consonant recognition.

To identify spectral and temporal cues for naturally produced consonants, Allen et al. collected consonant confusion matrices as a function of cutoff frequency for both low-pass filtering (LPF) and high-pass filtering (HPF) schemes, time truncation from an onset of consonants, and signal-to-noise ratio (SNR) in normal-hearing (NH) listeners (Phatak and Allen, 2007; Phatak et al., 2008; Li and Allen, 2011; Li et al., 2012)Phatak, Lovitt, and Allen. Through the analysis of confusion matrices, they were able to identify frequency and time ranges for each consonant, which resulted in a significant positive change in recognition and labeled them as “target frequency and time ranges.” They also noticed specific frequency and time ranges that produced a significant negative change in recognition called “conflicting frequency and time ranges.” In phonetics, a “conflicting” cue is an acoustic property that is phonetically inconsistent with another acoustic property in the same utterance (e.g., a stop consonant with a long voice onset time (VOT) but low fundamental frequency onset in the following vowel or *vice versa*). In this article, “conflicting” frequency and time ranges were defined as the ranges that generate more consonant confusion than enhancement.

To enhance consonant recognition with the target and conflicting frequency and time ranges, Allen et al. used a novel signal processing tool called the Articulation Index-Gram (AI-Gram) (Li and Allen, 2011; Li et al., 2012). The AI-Gram comprises the combined use of the articulation index model (French and Steinberg, 1947; Allen, 2005) and the linear peripheral cochlear model (Li et al., 2010). To determine whether consonant recognition was affected by the target and conflicting frequency and time ranges, they conducted two pilot studies with NH listeners. Two stop consonants (/ka, ga/) were tested in noise with a 6- and 12-dB gain on the target frequency and time ranges and complete removal of the conflicting frequency and time ranges (Li and Allen, 2011). In another study, they tested four stop consonants (/ta, ka, da, ga/) in noise with a 6-dB gain, 6-dB attenuation, complete removal of the target ranges, and an unprocessed control condition (Kapoor and Allen, 2012). The results obtained from these two studies indicated that the additional gain on the target ranges and the removal of conflicting ranges enhances the consonant perception by 10–70%. These findings led to our prediction that a greater enhancement for other consonants can be achieved if the target ranges are intensified while conflicting ranges are removed.

One challenging aspect of the two pilot studies conducted by Li and Allen (2011) and Kapoor and Allen (2012) is that their analyses were primarily based on the subjects’ responses to consonant syllables produced by a few selective (good) talkers, which resulted in clearly spoken stimulus tokens. The use of the clearly spoken tokens may contribute to a higher benefit (min. 10% to max. 70%) of the AI-Gram processing in consonant recognition. It is known that clearly spoken consonants yield intelligibility advantages of 3–38% points relative to normal conversational speech for NH listeners in noise or reverberation (Helfer, 1997; Ferguson and Kewley-Port, 2002; Ferguson, 2004). Underlying the reasons for the intelligibility advantages of clearly spoken consonants include enhanced acoustic cues such as the following: overall longer durations, longer VOT for voiceless stops, and increased consonant–vowel amplitude ratio (CVR) for stops and some fricatives (Chen, 1980; Picheny et al., 1986). Thus, it is unclear whether the significant consonant enhancement reported in Allen’s studies is due to AI-Gram processing on the target and conflicting cues or the combined effects with the use of the clearly spoken consonants produced by highly selective talkers. Another factor in Allen’s pilot studies (Li and Allen, 2011; Kapoor and Allen, 2012) to further consider is that, for each consonant, the averaged target and conflicting ranges over the NH subjects were used. By using the averaged target and conflicting frequency and time ranges, the potential for an intersubject variation in the target and conflicting ranges is not accounted. An intersubject variation in the target and/or conflicting ranges might not be a major issue for NH listeners but should be a critical factor for listeners with different configurations and degrees of hearing loss (Revoile et al., 1982; Ferguson and Kewley-Port, 2002).

In summary, previous studies demonstrate some efforts to determine the effect of the AI-Gram processing on consonant enhancement for four stop consonants. However, a more comprehensive assessment that includes the control of important confounding variables (conversationally spoken consonants and the use of tailored target and conflicting ranges) is warranted. In this study, using conversationally produced consonants by a single female talker and the target and conflicting frequency and time ranges identified from each NH subject, two experiments with NH listeners were conducted to determine how both the target and conflicting frequency and time ranges affected consonant enhancement when combined. The ideal approach for both experiments is to make direct comparisons between the results gained with conversationally and clearly spoken stimulus tokens, along with acoustical differences between the speech samples. Instead, extensive comparisons were made between the current study and Allen et al.’s studies (Li and Allen, 2011; Kapoor and Allen, 2012) in the “Discussion” section as the same AI-Gram was used to intensify and remove the target and conflicting ranges of the similar sets of consonants. In the first experiment, the target and conflicting frequency and time ranges for each consonant were identified on an individual listener basis. In the second experiment, the effects of the AI-Gram processing on the target and conflicting frequency and time ranges on consonant enhancement were determined.

## Experiment 1: Identify Target and Conflicting Frequency and Time Ranges

### Materials and Methods

#### Subject

Thirteen NH adults (seven women and six men; aged 19–43 with an average age of 28 years old) participated. All participants were native American English speakers and had thresholds better than a 20-dB hearing level at audiometric frequencies ranging from 0.25 to 8 kHz. All participants had interaural thresholds less than a 10-dB hearing level. All subjects provided informed consent, and all procedures were approved by the Texas Tech University Health Sciences Center Institution Review Board.

#### Stimuli

Stimuli included 14 frequently used consonants with the common vowel /a/ (/pa, ba, ta, da, ka, ga, ma, na, fa, va, sa, za, ʃa, ʒa/) in American English (Hayden, 1950). This study chose to test these consonants so that the results of this study could be directly compared to the results found in Allen’s studies (Li and Allen, 2011; Kapoor and Allen, 2012). To obtain conversationally spoken stimuli, each consonant syllable was produced by a single female with three different speaking efforts: minimum, medium, and maximum. For the minimum speaking effort, the talker was instructed to speak as if she was speaking to one NH listener in a quiet room. For the medium speaking effort, she was instructed to speak as she would to an NH listener in an everyday conversation, and for the maximum speaking effort, she was instructed to speak as if she was talking to a person with a hearing loss (Ferguson and Morgan, 2018). There were 42 sound files (14 consonants × 3 speaking efforts). All sound files were resampled from their original recorded sampling rate of 22,050–44,100 Hz, which is the standard for most consumer audio, and then normalized to have the same long-term root mean square energy [65 dBA sound pressure level (SPL)].

To choose one “conversationally spoken” token per consonant syllable, five lab members (all female students with an average age of 24 ± 1.6 years) were asked to rate how clearly the sound was spoken. They had normal hearing, which was verified through a pure-tone audiometry test. Each of the 42 sound files was randomly presented five times in quiet *via* headphones, and each lab member was asked to rate “how clearly the sound was spoken” on a scale from 1 to 7: 1—lowest possible clarity, 2—very unclear, 3—somewhat unclear, 4—midway, 5—somewhat clear, 6—very clear, and 7—highest possible clarity (Ferguson and Morgan, 2018). The lab members made their selections by clicking on the desired category from the graphical user interface and then pressing the “next” key to continue. The lab members were instructed to use the whole 1–7 scale and to focus on how clearly the sound was spoken with reference to the sounds they heard that day instead of speech heard in the past. For each consonant syllable, 75 ratings were collected (i.e., 3 different speaking efforts × 5 repetitions × 5 lab members). The sound file with the median rating was chosen as a “conversationally spoken” syllable. It turned out that all conversationally spoken stimuli used for the experiments resulted in consonant phonemes recorded with medium speaking efforts. All other tokens were not used for the experiment. The acoustic analyses on the final conversationally spoken stimuli showed an average fundamental frequency of 228 Hz. Complete silent parts were manually removed, which were identified by looking at waveforms and spectrograms, from both the onset and offset of the consonant syllables. Each processed consonant syllable was presented five times in quiet to all the five lab members. The processed consonant syllables were accepted as stimuli if they were perceived at a level of 99% correct, averaged over five presentations. The average duration plus SD of consonants was 406.57 ± 102.61 ms. The duration of each consonant is given in Table 1.

**TABLE 1:** The lower and upper ranges of the target and conflicting frequency and time.

Consonants (duration in ms)	Onset of vowel (ms)	Target frequency (kHz)		Conflicting frequency (kHz)		Target time (ms)	
		Lower ranges	Upper ranges	Lower ranges	Upper ranges	Lower ranges	Upper ranges
/pa/ (240)	59	0.3–0.8	7.1–7.4	1.1–1.7	2–2.2	8–18	40–60
		SD+: 4, SD−: 1		SD+: 2, SD−: 4		SD+: 3, SD−: 2	
/ba/ (331)	32	0.3–1.1	4–4.5	0.6–1.2	1.8–2.5	8–15	25–30
		SD+: 4, SD−: 2		SD+: 4, SD−: 2		SD+: 3, SD−: 3	
/ta/ (338)	96	3–3.7	5–7.4	1.3–1.7	2.2–2.8	26–50	42–70
		SD+: 2, SD−: 3		SD+: 3, SD−: 3		SD+: 2, SD−: 2	
/da/ (240)	43	3–4.1	6–7.8	1.1–1.7	2.3–2.8	15–24	25–31
		SD+: 3, SD−: 3		SD+: 2, SD−: 3		SD+: 2, SD−: 3	
/ka/ (447)	100	0.9–1.4	2–2.5	5–5.7	7.2–7.8	50–65	70–80
		SD+: 4, SD−: 4		SD+: 3, SD−: 4		SD+: 3, SD−: 4	
/ga/ (348)	52	1–1.5	1.6–2	3.4–4	4.8–5.2	21–31	45–52
		SD+: 1, SD−: 2		SD+: 5, SD−: 2		SD+: 5, SD−: 2	
/ma/ (350)	112	0.3–0.6	0.8–1.3	1.2–1.6	2–2.5	38–51	65–85
		SD+: 3, SD−: 5		SD+: 3, SD−: 1		SD+: 1, SD−: 1	
/na/ (400)	107	1–1.8	1.8–2.4	0.4–0.6	0.8–1.3	22–30	71–80
		SD+: 3, SD−: 2		SD+: 3, SD−: 4		SD+: 2, SD−: 3	
/fa/ (548)	180	0.4–0.7	2–2.7	3–3.6	7.4–7.8	45–65	82–90
		SD+: 4, SD−: 3		SD+: 2, SD−: 4		SD+: 3, SD−: 3	
/va/ (349)	88	0.4–0.7	1.2–1.7	1.2–1.7	4.2–4.8	25–49	50–70
		SD+: 2, SD−: 4		SD+: 3, SD−: 3		SD+: 2, SD−: 3	
/sa/ (501)	202	3.9–5.8	7.4–7.8	3–3.6	4.5–5.5	70–100	148–175
		SD+: 4, SD−: 2		SD+: 2, SD−: 3		SD+: 2, SD−: 2	
/za/ (501)	197	3.1–4.6	7.2–7.8	3–3.8	4.2–5.4	40–55	75–95
		SD+: 2, SD−: 3		SD+: 2, SD−: 4		SD+: 2, SD−: 2	
/ʃa/ (549)	238	1.9–2.5	2.9–4.2	3.9–4.5	7.2–7.8	80–110	180–225
		SD+: 3, SD−: 3		SD+: 4, SD−: 3		SD+: 3 SD−: 2	
/ʒa/ (550)	260	1.4–2.6	2.9–3.9	5–5.6	7–7.8	30–45	65–80
		SD+: 1, SD−: 3		SD+: 2, SD−: 3		SD+: 2, SD−: 3	

*The number of subjects with one SD above and below the mean of the target and conflicting ranges is indicated by SD+ and SD−, respectively. The duration of each consonant and an onset of the vowel /a/ from the beginning of each consonant are also given. The target time ranges indicate the consonant temporal duration from an onset of the vowel /a/.*

#### Identifying Target and Conflicting Frequency Ranges

For the identification of the target and conflicting frequency ranges, consonant confusion matrices were first collected and each of the 14 consonant syllables in the matrices was diotically presented in quiet to both ears *via* circumaural headphones (Sennheiser HDA-200, OLd Lyme, CT, United States). All stimuli were presented at the most comfortable level (MCL, range: 50–70 dB SPL). To determine the listener’s MCLs, each subject was asked to rate the loudness of each of the 14 unprocessed consonants in quiet according to the Cox loudness rating scale (Cox et al., 1997). The MCL was the mean of dB SPLs, which were rated “comfortable.” The confusion matrices were collected under both HPF and LPF. Each subject responded by pressing 1 of the 14 response buttons labeled in a consonant- /a/ context on a computer screen. To acquire reliable confusion matrices for each filtering scheme, each consonant was presented five times. For each presentation, the same consonant was presented sequentially, and each of these five presentations was only considered correct if the consonant presented was selected three times in a row. The order of which the consonants were presented was randomized over the five presentations.

**FIGURE 1:**
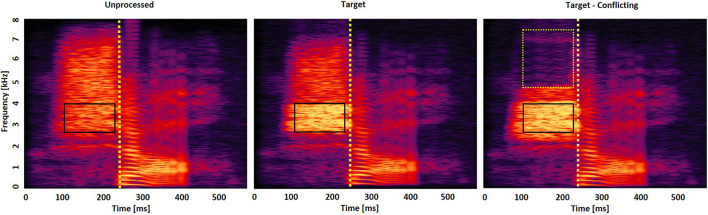
Spectrogram of /ʃa/ consonant under the three signal processing conditions: unprocessed **(left panel)**, intensified target frequency and time ranges by + 6 dB gain (middle panel), and the combined intensified target ranges and removed conflicting ranges **(right panel)**. Solid squares indicate the target frequency and time ranges, whereas the dotted square indicates the conflicting frequency and time ranges. Dotted vertical lines indicate an onset of the vowel /a/.

A reason to measure confusion matrices with both LPF and HPF is that the low and high frequencies may affect consonant perception in different ways (Miller and Nicely, 1955). For HPF, 7 kHz was used as an initial cutoff frequency to exclude mid-to-high spectral information (i.e., 3–7 kHz) known to be useful for some fricative consonant perception (Jongman et al., 2000). For LPF, 0.1 kHz was used as an initial cutoff frequency to include minimal spectral information for consonant perception (Dubno and Levitt, 1981). The step size for the cutoff frequency of both filtering schemes was 0.1 kHz. When a participant’s response was incorrect, the cutoff of the HPF was decreased by 0.1 kHz (i.e., from 7 to 6.9 kHz), and the cutoff of the LPF was increased by 0.1 kHz (i.e., from 0.1 to 0.2 kHz). The target frequency range was defined as the frequency range, in kHz, that provided the highest performance and was decreased 40% from that peak performance from both LPF and HPF conditions. For example, the recognition of /ʃa/ reaches 60% correct when the LPF cutoff is 2.1 kHz and reaches a maximum of 100% correct when the cutoff is moved from 2.1 to 2.2 kHz. So, the lower edge of the target frequency would be 2.1 kHz. When the HPF cutoff is 3.5 kHz, the recognition of /ʃa/ reaches a score of 60% correct and a maximum of 100% correct when the cutoff is moved from 3.5 to 3.4 kHz. So, the upper edge of the target frequency would be 3.5 kHz. Therefore, the final target frequency range would be 2.1–3.5 kHz (see the middle panel of Figure 1). About 20, 40, 60, and 80% below the peak performance were tested with five NH listeners. Decreasing 40% from the peak performance provided the highest benefit when the frequency range was boosted by 6 dB. Figure 1 displays the spectrograms of the unprocessed (left panel), intensified target frequency and time ranges by a 6-dB gain (middle panel), and the combined intensified target ranges with the removal of conflicting ranges (right panel) for /ʃa/. Squares indicate the target frequency and time ranges. Dotted vertical lines indicate an onset of the vowel /a/.

To identify the conflicting frequency ranges, confusing consonants that were consistently perceived two times above the chance level performance [i.e., 2^∗^(1/14)^∗^100 = 14.2% points] across the cutoff frequencies were determined. This criterion was intentionally selected to choose the top two or three major confused consonants. Then, for each confused consonant, the conflicting frequency range was defined as the frequency range in kHz that created the highest scores of the confused consonant (i.e., confusion or error, against the consonant presented) and 20% less than the peak error from both LPF and HPF conditions. For example, with the consonant /ʃa/ presented, the recognition of the confused consonant /sa/ reaches 24% correct when the LPF cutoff is 4.2 kHz and a maximum of 30% correct when the cutoff is moved from 4.2 to 4.3 kHz (i.e., 24% correct is 20% below the peak 30% error). Therefore, the lower edge of the conflicting frequency would be 4.2 kHz. When the HPF cutoff is 7.6 kHz, the recognition of the confused consonant /sa/ reaches a score of 24% correct and a maximum of 30% correct when the cutoff is moved from 7.6 to 7.5 kHz. So, the upper edge of the target frequency would be 7.6 kHz. Thus, the final conflicting frequency range would be 4.2–7.5 kHz for the recognition of the consonant /ʃa/ (see the right panel of Figure 1). A current pilot study with five NH listeners showed that decreasing 20% from the peak error provided the highest benefit. When this study tried with a decrease of 40% or more from the peak error, more consonant confusions emerged for most of the 14 consonants when these frequency ranges were removed. No trial-by-trial feedback was provided during the test. The complete test protocol, including breaks, took approximately 2.5 h per listener.

#### Identifying Target and Conflicting Time Ranges

We collected consonant confusion matrices by presenting each consonant diotically in quiet. The presentation of each consonant occurred five times to acquire reliable confusion matrices for each filtering scheme. The same consonant was presented sequentially and was considered correct only if selected three times in a row. The order of consonants presented was randomized over the five presentations. The initial duration of each consonant was 3% of the total duration from the onset (i.e., the remaining 97% of the consonant was truncated out) so that minimal consonant information was presented. The duration of the consonant was increased by 1 ms when a participant’s response was incorrect. The target time range was defined as a consonant segment in milliseconds from an onset of the vowel /a/ that provided the highest performance and was decreased 40% from that peak performance. A pilot study conducted by the author with five NH listeners showed that a 40% decrease from the peak provided the highest benefit when that range was boosted by 6 dB. The target time ranges were defined from the onsets of the vowel /a/ instead of defining them from the onsets of the consonants because the onsets of the vowel can be measured more reliably and precisely. An onset of the vowel /a/ was defined perceptually using a gating technique (’t Hart and Cohen, 1964). In this technique, short segments of speech were gated out from the consonant paired with the vowel /a/ and were presented in isolation. When the gate was slowly shifted in the direction of the vowel, there was a point in which one could begin to perceive an onset of the vowel. In this study, the detection of the vowel onset was verified by five adult NH listeners, and the vowel onset was accepted if all the five listeners agreed. If participants did not reach an agreement, the gating procedure were repeated with a 0.5-ms step on the segments that the five listeners disagreed upon. In Figure 1, the target time range for /ʃa/ is 89–193 ms, indicating a temporal duration of /ʃ/ from the onset (238 ms) of the vowel. The conflicting time ranges were not separately identified, but instead the conflicting time range was defined as the coincided target time range. A theoretical reason for the overlapped target and conflicting time ranges is that the conflicting frequency ranges would be the most detrimental factor to affect the target frequency ranges if they temporally coincide. No trial-by-trial feedback was provided during the test. The complete test protocol, including breaks, took approximately 1.5 h per listener.

### Results

**FIGURE 2:**
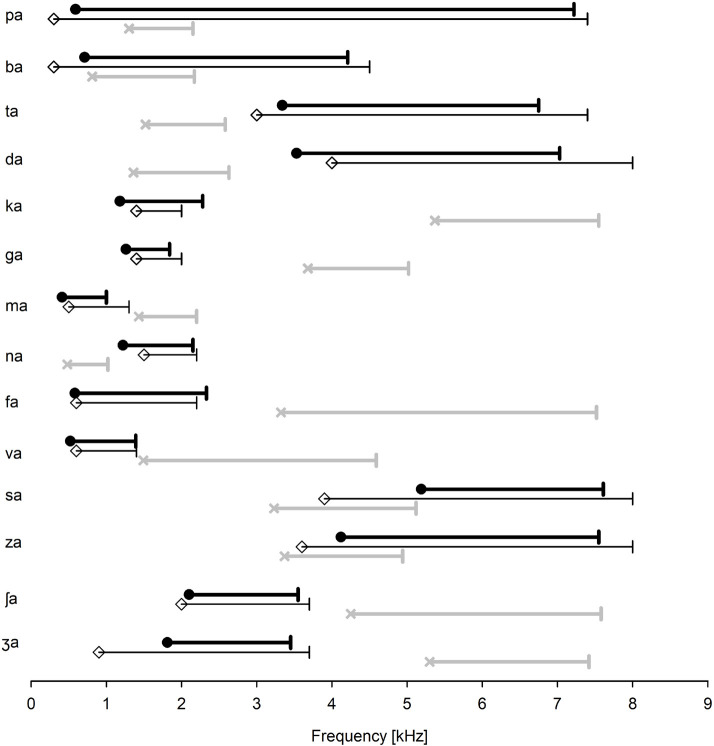
The mean target (filled circles) and conflicting (×symbols) frequency ranges for each consonant. Three consonants (/pa, ba, za/) have a partial or complete overlap between the target and conflicting frequency ranges. For comparison, the target frequency ranges, as reported by Li et al. (2010, 2012), are also presented (open diamond symbols).

The identification of the target and conflicting frequency and time ranges was administered for each listener, but the mean data was presented in this study. In Figure 2, the mean target (filled circles) and conflicting (×symbols) frequency ranges for each consonant were presented, along with the mean target frequency ranges (open diamond symbols) reported by Li et al. (2010, 2012). For the perception of the two bilabial stops (/pa/ and /ba/), wide ranges of spectral information were required. The target and conflicting frequency ranges fully overlapped. For the perception of the two alveolar stops (/ta/ and /da/), higher spectral ranges were required. The target frequency ranges were separated from the conflicting frequency ranges, but the conflicting frequency ranges were within similar ranges to each other. The perception of the two velar stops (/ka/ and /ga/) was dominated by mid spectral ranges. The conflicting frequency ranges were high and separated from the target frequency ranges. The perception of /ma/ and /na/ consonants required low and mid spectral ranges, respectively. The target and conflicting frequency ranges for both nasals did not overlap. The two labiodental fricatives (/fa/ and /va/) had similar lower edges of the target frequency ranges but different higher edges. Both had wide ranges of conflicting frequencies. The two alveolar fricatives (/sa/ and /za/) were characterized by high frication energy and were separated from all other consonants with high target frequency ranges above 4 kHz. Their conflicting frequency ranges had spectral energy at higher frequencies. The target and conflicting frequency ranges for /za/ were partially overlapped. For the two palatal fricatives (/ʃa/ and /ʒa/), the target frequency ranges resided at medium frication frequency energy. They were separated from all other consonants but were closer to each other, between which landed between 2 and 3 kHz. Their conflicting frequency ranges resided to be higher than 4 kHz. It should be noted that the three consonants /pa, ba, za/ had a full or partial overlap between the target and conflicting frequency ranges.

**FIGURE 3:**
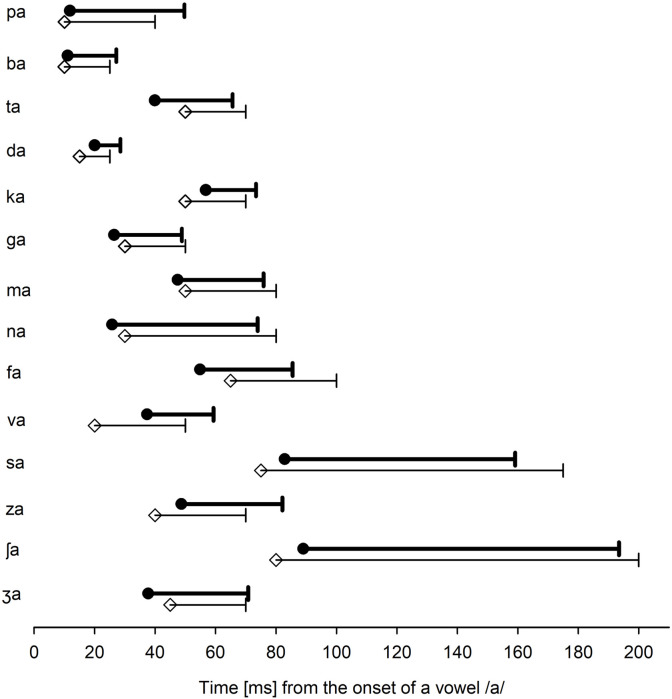
The mean target time ranges (filled circles) for each consonant. Note that the target time ranges indicate a temporal segment from an onset of the vowel /a/. For comparison, the target time ranges, as reported by Li et al. (2010, 2012), are also presented (open diamond symbols).

Figure 3 shows the mean target time ranges (filled circles), along with the mean target time ranges (open diamond symbols), reported by Li et al. (2010, 2012). Again, the conflicting time ranges were not separately identified. Instead, the target time ranges were used as the conflicting time ranges because the conflicting frequency ranges would be the most detrimental factor to affect the target frequency ranges if they temporally coincided. Here, the target time range was defined as the temporal duration of a consonant from the time point at the beginning of the vowel onset /a/. The target time ranges of the voiced and unvoiced stops do not overlap considerably, except for the bilabial /pa/ and /ba/ consonants. Target time ranges for voiced stops were shorter than the target ranges for unvoiced stops, except for velar consonants. The perception of /ma/ required a shorter target time (28.4 ms) than /na/ (48.2 ms), although the target time ranges partially overlapped between the nasals. Consonants /sa/ and /ʃa/ had the longest target time ranges (76.2 and 104.5 ms, respectively), which were well separated from all other consonants. Target time ranges for unvoiced fricatives (70.4 ms) were longer than those for voiced fricatives (29.5 ms).

Table 1 presents more detailed information, such as the lower and upper frequency as well as time ranges across subjects per each consonant. Table 1 also shows the number of subjects with one SD above and below the mean of the target and conflicting frequency ranges, indicated by SD+ and SD−, respectively. The number of subjects who had SD+ and SD− ranged from 3 to 8, depending on the consonant.

To compare the target frequency ranges obtained in this study with Li et al.’s data (open diamond symbols in Figure 2), a two-tailed independent *t*-test was conducted. The analysis showed no significant difference in the lower target frequency ranges, *t*(24) = 2.06, *p* = 0.78 and the upper target frequency ranges, *t*(24) = 2.06, *p* = 0.82. A two-tailed independent *t*-test also showed that the target time ranges were not statistically different from Li et al.’s (2010,2012) data (2010, 2012) for the lower, *t*(24) = 2.06, *p* = 0.87, or the upper ranges, *t*(24) = 2.06, *p* = 0.95. It should, however, be noted that many subjects who had SD + and SD− (see Table 1) indicated a large variability in the target frequency and time ranges.

### Discussion

Our results showed that the target and conflicting ranges are highly consonant-specific (Figures 2, 3). The mean data obtained in this study were comparable with Li et al.’s (2010,2012) data (2010, 2012); however, this study data showed that the target and conflicting ranges were highly listener specific (Table 1). Stop consonants are mainly characterized by a short-duration burst from the onset (e.g., 10–20 ms), by their center frequency (wide bank, high, and medium), and their formant (particularly F2) transitions (Delattre et al., 1955; Stevens and Blumstein, 1978; Blumstein and Stevens, 1979, 1980). In this study, The target frequency ranges for stop consonants are consistent with these typical acoustic cues. Bilabial stops had wideband clicks: 0.6–7.2 kHz for /pa/ and 0.7–4 kHz for /ba/. Alveolar stops had a high burst frequency: 3.3–6.8 kHz for /ta/ and 3.5–7 kHz for /da/. Medium burst spectral bands 1.2–2.3 and 1.3–1.8 kHz were target frequency ranges for velar stops /ka/ and /ga/, respectively. The two nasals are known to share a common feature of nasal murmur at low frequency but differed from each other in their F2 onset below 2.4 kHz (Kurowski and Blumstein, 1984; Repp, 1986; Ohde, 1994; Ohde and Ochs, 1996; Ohde et al., 2006). In this study, the results are consistent with this view: the perception of /ma/ and /na/ was dominated by low and mid spectral ranges 0.4–1.0 and 1.2–2.2 kHz, respectively.

Fricative consonants are considered a major source of perceptual error in consonant recognition in noise (Miller and Nicely, 1955; Hughes and Halle, 1956). Common characteristics of fricatives include spectral distribution of the frication noise (Hughes and Halle, 1956; Jongman et al., 2000), formant transition (Soli, 1981), overall amplitude (Behrens and Blumstein, 1988), and long duration (Baum and Blumstein, 1987; Jongman, 1989). It is known that labiodental fricatives (/f/ and /v/) show relatively flat spectra below 10 kHz with no dominating spectral peaks (McGowan and Nittrouer, 1988; Nittrouer, 2002). This may explain that non-sibilant fricatives /fa, va, θa, ða/ are involved in more than half of the confusions at 12-dB SNR in white noise (Phatak et al., 2008). In this study, the target frequency ranges have low frication frequency energy (0.6–2.3 kHz for /fa/ and 0.5–1.4 kHz for /va/) and are within similar ranges to those reported by Li et al. (2012).

Unlike the labiodental fricatives, the sibilant alveolar consonants /sa/ and /za/ and palatal consonants /ʃa/ and /ʒa/ are seldom confused with any other consonants at 12-dB SNR (Phatak et al., 2008). Spectral cues are well defined for both alveolar and palatal consonants. In this study, the alveolar consonants were characterized by high frication energy, 5.2–7.7 kHz for /sa/ and 4.1–7.6 for /za/. These ranges are comparable with known frequency cue ranges: 4–7.5 kHz (Li and Allen, 2011), above 4 kHz (Hughes and Halle, 1956), 3.5–5 kHz (Behrens and Blumstein, 1988), and 6–8 kHz (Jongman et al., 2000). Compared to alveolar consonants, the perception of palatal fricatives /ʃa/ and /ʒa/ is known to require a lower spectral peak around 2–3.5 kHz (Miller and Nicely, 1955) and 2–4 kHz (Hughes and Halle, 1956; Behrens and Blumstein, 1988). In this study, the target frequency ranges resided at a medium frication frequency energy: 2.1–3.6 kHz for /ʃa/ and 1.8–3.5 kHz for /ʒa/, all of which are comparable to the known frequency ranges.

Regarding the target time ranges, the mean duration of the target time ranges for unvoiced stops (26.7 ms) was longer than the mean duration for voiced stops (15.7 ms), which is comparable with the mean durations reported by Li et al. (2010). The mean durations of the target time ranges for /ma/ and /na/ were 28.4 and 48.2 ms, respectively. Li et al. (2010) also reported similar durations. The mean duration of the target time ranges for unvoiced fricatives (70.4 ms) was longer than that for voiced fricatives (29.5 ms), which is also comparable with the mean duration reported by Li et al. (2010). However, the target time ranges either barely or did not overlap between the voiced and unvoiced fricatives (Figure 2). Although the mean duration of unvoiced fricatives is generally longer than that of the voiced fricatives, the distribution of the two overlaps considerably (Baum and Blumstein, 1987). Labiodental fricatives (/fa/ and /va/) had a shorter duration of the frication noise region compared to sibilants (/s, ʃ, z, ʒ/), indicating that the duration of the frication noise is a primary parameter to distinguish sibilant consonants from non-sibilant consonants. This finding is consistent with the findings reported by Miller and Nicely (1955). Furui conducted a time truncation experiment with Japanese consonant–vowel syllables in NH listeners (1986). In the study, consonant recognition was measured as a function of truncation position, relative to the critical point at which the syllable identification scores exceeded 80% correct. They found that consonant recognition scores rapidly decreased from 90% correct to 30% correct when the truncation point passed through the critical point 20 ms away from the critical point. This study’s data, along with Furui’s data, suggest that a short interval including the maximum transition position, which can be related to the perceptual critical points and bears sufficient perceptual information for syllable identification.

There are a few limitations in Experiment 1. As common speech acoustics of consonants (duration, VOT, and CVR) are also highly talker dependent, it is expected that the target and conflicting frequency ranges are highly talker dependent (Mullennix et al., 1989; Allen et al., 2003; Magnuson and Nusbaum, 2007). The target and conflicting ranges also vary depending on the preceding and following vowels (Harris, 1958; Heinz and Stevens, 1961; Blumstein and Stevens, 1980; Jongman et al., 2000; Stilp, 2020). In addition, different noise levels may have significant effects on the target and conflicting ranges (Li et al., 2010). It is understood that a more realistic identification condition for the target and conflicting ranges should include these factors; this allows the results to be more generalized. In this study, an initial intention for this experiment was to focus on the use of our identification scheme in quiet with a single talker to determine the feasibility of the identification approach studied in this paper With the proof of feasibility, an identification scheme can then be developed to take in vowels and words produced by multiple talkers and as a function of SNR as well. Larger target and conflicting data sets that have been generated and will be generated could be used to achieve the long-term goal of this study: developing algorithms for artificial intelligence-powered signal processors.

## Experiment 2: Measure Consonant Enhancement

### Subjects and Stimuli

The same subjects who participated in Experiment 1 participated in this experiment as well. The 14 consonants used in Experiment 1 were used in Experiment 2, however, each consonant was processed by the AI-Gram: with target frequency and time ranges enhanced by + 6 dB gain (i.e., target) and with both the target ranges enhanced and conflicting ranges removed (i.e., target-conflicting). The processed consonants were accepted as stimuli if they were perceived by five lab members at a 99% correct level in quiet. For the three consonants (/pa, ba, za/) with overlapping target and conflicting frequency ranges (Figure 2), the target frequency ranges were intensified while the overlapped conflicting frequency ranges were not removed. The selection of a 6-dB gain was based on the pilot data with five NH listeners, suggesting that gains lower than 6 dB provided little or no consonant enhancement and that gains greater than 9 dB generated sound distortion.

### Procedure

Subjects were seated in a single-walled sound-treated booth (Industrial Acoustics Company, North Aurora, IL, United States). Before formal testing, a 30-min familiarization session (15-min each for the target and the target-conflicting) was binaurally provided. Consonant recognition was binaurally measured in noise at −30, −20, and −10 dB SNR (speech-weighed noise) under the three signal processing conditions: the unprocessed, target, and target-conflicting. The choice of these SNRs was based on a previous study with NH listeners (Yoon et al., 2019) and was used both to validate the benefits of the AI-Gram processing in noise and to avoid a ceiling effect. Speech-shaped noise was used because the information identifying individual phonemes occurs over a very short time frame, and it was reasoned that fluctuations presented in maskers might lead to undue variability in performance. This noise masker was combined with the unprocessed and AI-Gram processed consonants to generate the designated SNRs. The sum of the speech signal and masking noise was filtered with a band-pass filter of 100–8,500 Hz before presentation. This bandwidth included the target and conflicting frequency ranges for all consonant syllables. The overall presentation level of the band-pass filtered output (i.e., speech plus noise) was scaled to the subject’s MCLs (range: 50–70 dB SPL), assessed in Experiment 1. The masker commenced 500 ms before an onset of the target speech and continued for 500 ms after the target offset with cosine onset and offset ramps of 100 ms applied to the mixture. The combined speech and noise signal was diotically delivered *via* an audiometer (GSI AudioStar Pro, Eden Prairie, MN, United States) to Sennheiser HDA-200 circumaural headphones. Each consonant syllable was randomly presented ten times at each SNR. No trial-by-trial feedback was provided during the test. The complete test protocol (3 signal processing conditions × 3 SNRs × 14 consonants × 10 repetitions of each consonant), including breaks, took approximately 4 h per listener.

### Results

**FIGURE 4:**
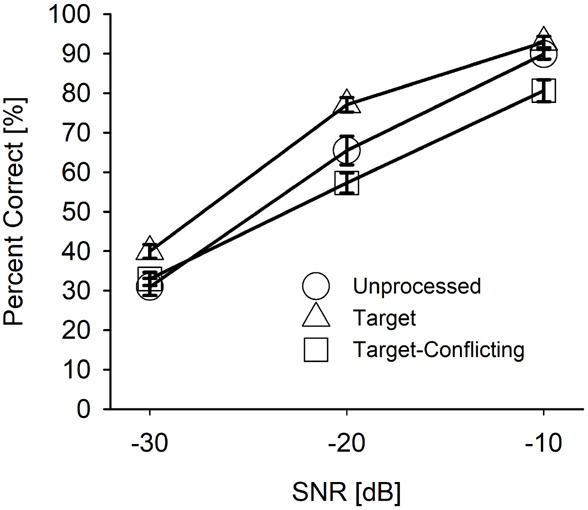
Mean percent correct scores with SEs for each signal processing condition as a function of signal-to-noise ratio (SNR).

**FIGURE 5:**
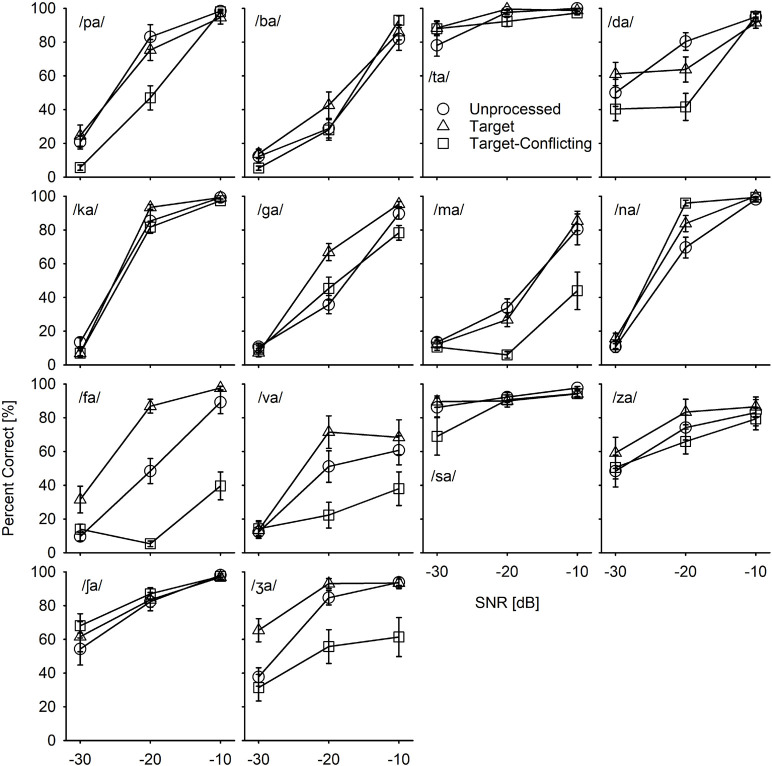
Mean consonant recognition scores for individual consonant as a function of SNR and of the signal processing.

Based on the mean (Figure 4) and individual consonant (Figure 5) analyses, there are two major findings: (1) consonant recognition improved the most with the target condition but deteriorated with the target-conflicting condition compared to the scores with the unprocessed condition and (2) the perception of seven consonants (/pa, da, ga, ma, fa, va, ʒa/) was significantly affected by signal processing, but the perception of the remaining seven consonants (/ba, ta, ka, na, sa, ʃa, za/) was not. The analyses presented in the following sections were performed with raw percent correct scores because the significance levels of the statistical analyses with transformed data sets (i.e., arcsin, log, or square root) for main effects remained unchanged, compared to the raw percent correct performances.

#### Mean Performance Data Analyses

Figure 4 depicts the mean percent correct with SEs for each signal processing condition as a function of SNR. Two-way repeated measure ANOVA showed a significant main effect of the signal processing, *F*(2,36) = 20.68, *p* < 0.001 and of the SNR, *F*(5,36) = 498.53, *p* < 0.001. Significant interactions between the signal processing and SNR were also observed, *F*(4,36) = 9.23, *p* < 0.001. All pairwise multiple comparisons with Bonferroni correction showed that comparisons between any two signal processing conditions were significant (*p* < 0.05), except for three comparisons: unprocessed vs. target (*p* = 0.96) at −10 dB SNR, unprocessed vs. target-conflicting (*p* = 0.66) at −30 dB SNR, and target vs. target-conflicting (*p* = 0.17) at −30 dB SNR. Multiple comparisons also showed a significant difference between any two SNRs (*p* < 0.001).

#### Individual Consonant by Consonant Analyses

To determine whether the effect of the signal processing on consonant recognition is consonant dependent, recognition scores were plotted with SEs per consonant (Figure 5). Table 2 presents the results of the two-way repeated measures ANOVA. The results showed that the main effects of the signal processing were highly consonant-specific. Perception of seven consonants (/pa, da, ga, ma, fa, va, ʒa/) was significantly affected by the signal processing, as indicated by bold *p*-values, while the perception of the other seven consonants (/ba, ta, ka, na, sa, ʃa, za/) was not significantly affected by the signal processing. The perception of all 14 consonants was also significantly affected by SNRs. A significant interaction was observed for seven consonants (/pa, da, ga, ma, na, fa, va/).

**TABLE 2:** Results of two-way ANOVA for each consonant with two factors: signal processing and signal-to-noise ratio (SNR).

	Signal processing	SNR	Interactions
/pa/	*F*(2,36) = 11.5, *p* < **0.001**	*F*(2,36) = 114.1, ***p* < 0.001**	*F*(4,36) = 4.8, ***p* = 0.003**
/ba/	*F*(2,36) = 1.1, *p* < 0.35	*F*(2,36) = 192.0, ***p* < 0.001**	*F*(4,36) = 2.2, *p* = 0.09
/ta/	*F*(2,36) = 0.4, *p* = 0.80	*F*(2,36) = 18.5, ***p* < 0.001**	*F*(4,36) = 1.3, *p* = 0.30
/da/	*F*(2,36) = 4.1, ***p* < 0.04**	*F*(2,36) = 73.1, ***p* < 0.001**	*F*(4,36) = 6.9, ***p* < 0.001**
/ka/	*F*(2,36) = 1.3, *p* < 0.31	*F*(2,36) = 562.8, ***p* < 0.001**	*F*(4,36) = 1.9, *p* = 0.14
/ga/	*F*(2,36) = 11.4, ***p* < 0.001**	*F*(2,36) = 203.3, ***p* < 0.001**	*F*(4,36) = 8.6, ***p* < 0.001**
/ma/	*F*(2,36) = 19.8, ***p* < 0.001**	*F*(2,36) = 213.1, ***p* < 0.001**	*F*(4,36) = 9.4, ***p* < 0.001**
/na/	*F*(2,36) = 3.7, *p* = 0.06	*F*(2,36) = 402.9, ***p* < 0.001**	*F*(4,36) = 4.5, ***p* = 0.005**
/fa/	*F*(2,36) = 30.0, ***p* < 0.001**	*F*(2,36) = 100.0, ***p* < 0.001**	*F*(4,36) = 16.6, ***p* < 0.001**
/va/	*F*(2,36) = 5.0, ***p* = 0.02**	*F*(2,36) = 29.1, ***p* < 0.001**	*F*(4,36) = 7.3, ***p* < 0.001**
/sa/	*F*(2,36) = 0.9, *p* = 0.39	*F*(2,36) = 9.1, ***p* = 0.002**	*F*(4,36) = 1.7, *p* = 0.17
/za/	*F*(2,36) = 1.1, *p* = 0.36	*F*(2,36) = 15.3, ***p* < 0.001**	*F*(4,36) = 0.5, *p* = 0.80
/ʃa/	*F*(2,36) = 0.4, *p* = 0.67	*F*(2,36) = 18.8, ***p* < 0.001**	*F*(4,36) = 0.7, *p* = 0.61
/ʒa/	*F*(2,36) = 8.5, ***p* = 0.003**	*F*(2,36) = 49.0, ***p* < 0.001**	*F*(4,36) = 2.0, *p* = 0.11

*A main effect of the two factors and their interactions were given for each consonant. Significant main effect was indicated by bold p-values.*

The pairwise multiple comparisons analysis with Bonferroni correction were administered for the seven consonants (/pa, da, ga, ma, fa, va, ʒa/) which were observed for a significant main effect of the signal processing. Only pairs with significant differences were presented with a significance level in Table 3. The results showed significant differences between the target and the target-conflicting conditions for all seven consonants over SNRs except for four consonants at a few specific SNRs between the unprocessed and the target condition. Significant differences between unprocessed and the target-conflicting conditions were observed for five consonants at a specific SNR.

**TABLE 3:** Pairwise multiple comparisons between the signal processing conditions for the seven consonants, which were observed for a significant main effect of the signal processing.

Consonants	At SNR	Unprocessed vs Target	Unprocessed vs Target-conflicting	Target vs Target-conflicting
/pa/	−30 dB			**
	−20 dB		***	***
/da/	−30 dB			*
	−20 dB	***		*
/ga/	−20 dB	***		***
	−10 dB		**	**
/ma/	−20 dB		***	**
	−10 dB		***	***
/fa/	−20 dB	***		***
	−10 dB		***	***
/va/	−20 dB	*		***
	−10 dB			*
/ʒa/	−30 dB			*
	−20 dB		*	**
	−10 dB		**	**

***p < 0.001, **p < 0.01, and *p < 0.05. Empty cells indicate “not significant.”

#### Confusions Pattern Analyses

One of the goals for this current study was to define the nature of consonant enhancement or loss evoked by the AI-Gram processing on the target and conflicting ranges. In this section, key details were provided on how consonant recognition was affected by different signal processing conditions using confusion matrix analyses. Based on the statistical analyses on individual consonants (Figure 5), confusion patterns were presented for two exemplary consonants from the seven consonants (/pa, da, ga, ma, fa, va, ʒa/) with a significant signal processing effect and the other seven consonants (/ba, ta, ka, na, sa, za, ʃa/) with a non-significant signal processing effect. The confusion patterns for other consonants are available in the Supplementary Material. For these figures of confusion pattern analyses, each signal processing condition is given as a title above each panel. The consonant presented is given at the top-left corner in the left panel, and all dependent values were plotted on a logarithmic scale for a better visualization of the confused consonants as a function of SNR. The percent scores for the consonant presented are denoted as a thick curve, whereas the percent scores for the confused consonants (competitors) are indicated as a thin curve with labels. Only the top three competitors are shown to avoid the congested figures.

**FIGURE 6:**
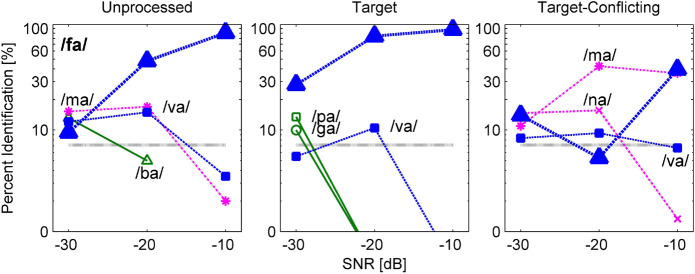
Confusion patterns when /fa/ was presented. Each signal processing condition is given as a title above each panel. The presented consonant is given in the left panel, and all dependent values are plotted on a logarithmic scale as a function of SNR. The percent correct scores for the presented consonant are denoted as a thick curve, whereas the percent scores for each non-presented consonant or competitors are indicated as a thin curve with labels. Only up to top three competitors are shown for a better visualization of the confusion patterns.

**FIGURE 7:**
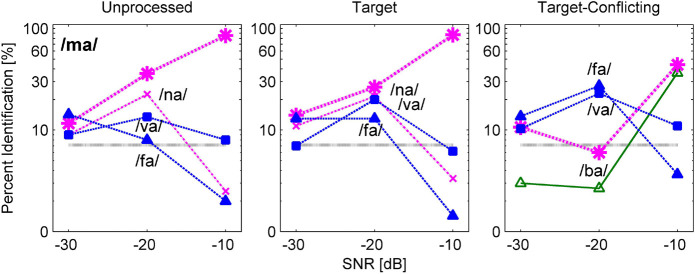
Confusion patterns when /ma/ was presented. A description for the figure is the same as presented in Figure 6.

Figures 6 and 7 are examples of significant effects of the AI-Gram signal processing on consonant recognition. Figure 6 shows the confusion patterns when /fa/ was presented. Percent scores of /fa/ were higher with the target condition but lower with the target-conflicting condition compared to scores with the unprocessed condition. With the unprocessed condition (left panel), the combination of three competitors /ba, ma, va/ led to more than 40% errors at −30 dB SNR and continued to compete with more than 30% errors at −20 dB SNR. With the target condition (middle panel), the percent scores of /fa/ improved at the lower two SNRs compared to the unprocessed because the confusion from /ma/ and /va/ was reduced. With the target-conflicting condition (right panel), the performance of /fa/ fell to below 20% correct at the two lower SNRs because of increasing confusions with two nasals. Based on these patterns, recognition enhancement with the target condition was due to reduced confusions from /ma/, whereas recognition deterioration with the target-conflicting condition was due to increased confusion from /ma/. Figure 7 shows the confusion patterns when /ma/ was presented. Percent scores between the unprocessed and the target condition were not statistically different, but percent scores with the target-conflicting condition were significantly lower from those with either the unprocessed or the target condition (see Table 3). Specifically, for both the unprocessed and the target condition, the three consonants /na, va, fa/ competed with /ma/ across SNRs. For the target-conflicting condition, the confusions from /fa/ and /va/ increased at −20 dB SNR and new confusions emerged from /ba/ at −10 dB SNR, resulting in a large decrease in /ma/ perception. Based on these results, the target condition did not help to improve the perception of /ma/. The target-conflicting condition added more confusions rather than enhancement.

The results of confusion analyses for the other five consonants (/pa, da, ga, va, ʒa/) are provided in the Supplementary Material. Like the confusion patterns for two consonants presented above, major competitors were similar across the signal processing condition, but confusion patterns were somewhat dependent on the signal processing. The confusions were reduced or resolved with the target condition, contributing to consonant enhancement. In contrast, the confusions were increased with the target-conflicting condition, leading to poorer consonant recognition compared to the unprocessed condition.

**FIGURE 8:**
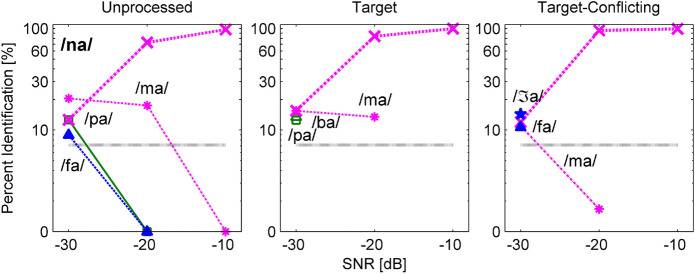
Confusion patterns when /na/ was presented. A description for the figure is the same as presented in Figure 6.

**FIGURE 9:**
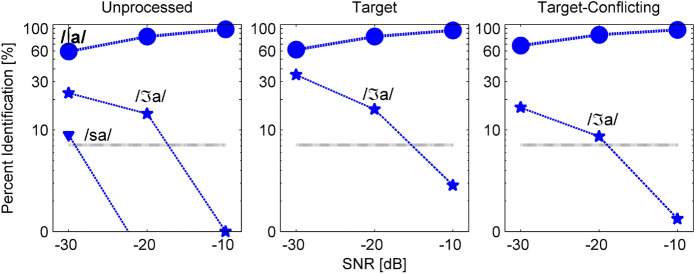
Confusion patterns when /ʃa/ was presented. A description for the figure is the same as presented in Figure 6.

Figures 8, 9 are examples of non-significant effects of the AI-Gram signal processing on consonant recognition. The two consonants /na/ and /ʃa/ were intentionally chosen because their percent scores were the highest with the target-conflicting condition even though these differences were not statistically significant compared to the scores with the other two signal processing conditions (Table 3). When /na/ was presented, one major and consistent competitor /ma/ emerged across the signal processing condition and SNR (Figure 8). With the target-conflicting condition, the confusions were resolved, leading to a rise in the identification of /na/ to near-perfect scores at the higher two SNRs. When /ʃa/ was presented, /ʒa/ competed across the signal processing and SNR (Figure 9). Compared to the two other conditions, the confusions were reduced with the target-conflicting condition, resulting in an improved perception at the lower two SNRs. Based on these results, the target-conflicting condition contributed to the enhancement of recognition for at least these two consonants even though their contribution was not statistically significant.

As presented in the Supplementary Material, the major competitors for each of the other five consonants (/ba, ta, ka, sa, za/) were similar across the signal processing conditions, and the confusion patterns were different. For example, the perception of the three consonants (/ba, sa, za/) improved with the target condition but decreased with the target-conflicting condition even though these changes in percent scores were not statistically significant. However, percent scores for /ta/ and /ka/ were similar across the signal processing condition and SNR.

#### Acoustic Features Analyses

**FIGURE 10:**
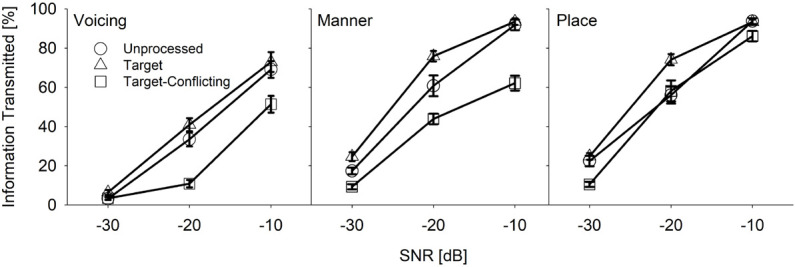
Mean percent information transmitted for voicing, manner, and place as a function of SNR and of the signal processing.

To determine which acoustic features (voicing, manner, and place) of consonants contributed to either consonant enhancement or loss, the percent information transmitted using information theory equations was computed (Wang and Bilger, 1973). Firstly, consonant syllables were categorized in terms of voicing, manner, and place features and then the percent correct for each of the three feature-based group consonants was computed. To obtain the percent information transmitted, the percent correct was divided by the total number of consonant syllables presented and then multiplied by 100. The results of these computations with SEs are shown in Figure 10. For voicing (left panel), two-way repeated measures ANOVA showed a significant main effect of the signal processing, *F*(2, 36) = 21.7, *p* < 0.001 and of SNR, *F*(2, 36) = 198.7, *p* < 0.001. The interaction effect was also significant, *F*(4, 36) = 12.3, *p* < 0.001. Mean manner information transmitted (middle panel) was significantly different over the signal processing, *F*(2, 36) = 30.2, *p* < 0.001 and SNR, *F*(2, 36) = 437.9, *p* < 0.001. Interaction was also significant, *F*(4, 36) = 9.3, *p* < 0.001. Mean place information transmitted (right panel) was significantly different over the signal processing, *F*(2, 36) = 12.3, *p* < 0.001, and SNR, *F*(2, 36) = 609.8, *p* < 0.001. Interaction was significant as well, *F*(4, 36) = 3.6, *p* = 0.02.

The results of pairwise multiple comparison analysis with the Bonferroni correction for each feature are given in Table 4 for the signal processing factor at each SNR. The analyses showed two main findings. The target condition had helped to transmit significantly improved information of manner and place at −20 and −10 dB SNR, compared to the two other conditions. In contrast, the target-conflicting condition significantly damaged the transmission of voicing and manner information at −20 and −10 dB SNR, compared to the other two conditions. For the SNR factor, a significant difference was observed for voicing between any two SNRs (*p* < 0.001) except for −30 vs. −20 dB SNR for the target-conflicting condition (*p* = 0.20). A significant difference was also observed for both manner and place cues between any two SNRs (*p* < 0.001). Based on these results, consonant perception was enhanced under the target condition through the improved transmission of manner and place cues while consonant perception was declined under the target-conflicting condition through the decreased transmission of voicing and manner cues.

**TABLE 4:** Pairwise multiple comparisons between the signal processing conditions for voicing, manner, and place.

Feature	At SNR	Unprocessed vs Target	Unprocessed vs Target-conflicting	Target vs Target-conflicting
Voicing	−20 dB		***	***
	−10 dB		***	***
Manner	−30 dB			*
	−20 dB	***	**	***
	−10 dB		***	***
Place	−30 dB			**
	−20 dB	***		***

***p < 0.001, **p < 0.01, and *p < 0.05.

### Discussion

Our mean percent correct data (Figure 4) reveals that consonant recognition improved the most with the target condition but deteriorated with the target-conflicting condition, compared to the scores with the unprocessed condition. Individual consonant analysis (Figure 5) showed that the perception of seven consonants (/pa, da, ga, ma, fa, va, ʒa/) was significantly affected by the signal processing, but the perception of the remaining seven consonants (/ba, ta, ka, na, sa, ʃa, za/) was not. Confusion analyses (Figures 6–9) showed similar competitors across the signal processing for each of all 14 consonants, but confusion patterns varied. Overall, the target condition had helped to reduce the confusions, resulting in improved consonant recognition, whereas the target-conflicting condition increased confusions, resulting in poorer consonant recognition compared to the unprocessed condition. Feature analyses (Figure 10) showed that consonant enhancement with the target condition was primarily attributed to better transmission of manner and place information. Consonant deterioration with the target-conflicting condition was primarily due to a poorer transmission of voicing and manner information. These results suggest that intensifying the target ranges is an effective way to improve consonant recognition in noise while removing the conflicting ranges negatively impacts consonant recognition.

#### Effects of the Signal Processing on Mean Consonant Recognition

Mean consonant enhancement was 9% points at −30 dB SNR, 11.6% points at −20 dB SNR, and 3% points at −10 dB SNR with the target condition, compared to the scores with the unprocessed condition (Figure 4). Under the target-conflicting condition, consonant recognition deteriorated, with 8.2% points at −20 dB SNR and 9.2% points at −10 dB SNR, compared to the scores with the unprocessed condition. These findings are different from the results reported in Kapoor and Allen’s (2012) study, which assessed the effects of the target condition on four stop consonants (/ta, da, ka, ga/) with 21 NH listeners over −12, −6, 0, +6, +12 dB SNR (speech-weighted noise). They reported a range of 10–70% points benefit of the target condition at SNR ≤ 0 dB, but approximately less than 7% points at SNR ≥ + 6 dB. Direct comparisons between the two studies should not be made even though the same AI-Gram processing was employed for consonant recognition. Potential reasons for these discrepancies between the two studies are discussed in a separate subsection below.

#### Effect of the Signal Processing on Individual Consonant Recognition

One of the major findings was that the perceptions of 12 out of 14 consonants (except for /na/ and /ʃa/) worsened under the target-conflicting condition (Figure 5). This finding is different from the result of Li and Allen’s (2011) study (2011), which measured consonant recognition with two stops /ga/ and /ka/ at −9 and −3 dB SNR (speech-weighted noise) with three NH listeners. They tested the four signal processing conditions: unprocessed, conflicting alone (i.e., complete removal of the conflicting ranges), and two target-conflicting (i.e., complete removal of the conflicting ranges and 6 or 12 dB gain on the target ranges together). They observed the two main trends. The first trend was that the effect of the conflicting condition alone was very different between /ga/ and /ka/. Compared to the scores with the unprocessed condition, the perception of /ga/ improved by 15.9 and 14% points at −9 and −3 dB SNR, respectively, while perception for /ka/ deteriorated by 19.2 and 42.8% points. A second trend was that the target-conflicting condition had helped to improve the perception of both /ga/ and /ka/ with a range of 3.6–36.9% points over SNR compared to the scores with the unprocessed condition. As Li and Allen (2011) did not measure the effect of the target condition alone, it is unclear whether the benefit of the target-conflicting condition stemmed from the target condition alone or not. The effect of the target condition was measured, and consonant enhancement for the seven consonants was demonstrated. When the combined effect of the target and the conflicting ranges was measured, consonant perception for the 12 consonants declined (some of them were significant while others were not). The data suggest that the conflicting condition alone negatively impacted consonant recognition.

There are three possible explanations for the different outcomes between the current study and the Li and Allen’s (2011) study. Firstly, the use of different SNRs may be a factor. For the current study, consonant confusions were measured at −30, −20, and −10 dB SNR (speech-weighed noise). Li and Allen collected consonant confusions at −9 and −3 dB SNR (speech-weighed noise). Secondly, the current study measured consonant confusions with 14 alternative-forced choices, whereas Li and Allen measured confusions with 6 alternative-forced choices. Finally, the target and the conflicting ranges were used, which were identified from each subject, whereas Li and Allen used averaged target and conflicting ranges. One or any combination of these factors could contribute to the differences in percent scores and perceptual confusions.

#### Acoustic Features

In terms of acoustic features, consonant enhancement with the target condition was primarily due to improved transmission of manner and place information. Consonant deterioration with the target-conflicting condition was primarily due to a poorer transmission of voicing and manner information. To show the overall effect of the AI-Gram signal processing, the transmitted percent information were presented, which was averaged across SNRs. The percent information with the target condition was better transmitted with 4.9, 8.1, and 6.7% for voicing, manner, and place, respectively, compared to the unprocessed condition. These differences were not statistically significant at most of the SNRs (see Table 4). This data are comparable with the Yoon et al. (2019) study, which measured the information transmitted for the same three features with the unprocessed and the target conditions. They observed the benefit of the target condition with 3%, 10%, and 6.5% for voicing, manner, and place compared to the unprocessed condition. The percent information transmitted was also averaged over the SNR. The current study also showed that the target condition had helped to transmit more voicing (18.3%), manner (26.3%), and place (12.5%) compared to the target-conflicting condition. In contrast, the target-conflicting condition damaged the transmission of voicing (13.4%), manner (18.2%), and place (5.8%), compared to the unprocessed condition. These differences were statistically significant at most of the SNRs (see Table 4). No comparable data exist; direct comparisons cannot be made.

One clear trend is that the transmission of manner information was mostly enhanced with the target condition but most damaged with the target-conflicting condition. For consonants, the size of the constriction provides a clue to the manner. The first formant (F1) is known to be most affected by the size of the vocal tract constriction (Stevens and Klatt, 1974; Soli, 1981). The results of this study that F1 was enhanced with the target condition but worsened with the target-conflicting condition. It is known that the second formant frequency (F2) and F2 transition are important acoustic cues to the place of articulation for consonant recognition (Kurowski and Blumstein, 1984). A reason for the improved transmission of place information with the target condition may be due to enhanced F2 and F2 transition.

#### Limitations

One specific concern is the possibility that the optimal effect may occur with the target-conflicting condition if different levels of attenuations are applied to the conflicting ranges, as opposed to complete removal. This possibility is supported by the finding that consonant recognition (12 out of 14 consonants) worsened with the target-conflicting condition. In addition, in a pilot study with individuals with hearing aids or cochlear implants, maximum consonant enhancement was observed when the conflicting ranges were attenuated by −6 dB. It had been also found that attenuations between −6 and −20 dB did not make any significant difference in consonant enhancement. Our data and the pilot data with device users suggest that intensified target and attenuated conflicting ranges may facilitate a greater consonant enhancement.

#### Future Plan and Clinical Application

Our long-term plan is to develop an individually customized fitting scheme using an artificial intelligence-powered algorithm for bimodal users, who wear a hearing aid in one ear and a cochlear implant in the opposite ear. The current audiogram-based bimodal fitting provides highly mixed outcomes (Dorman and Gifford, 2010; Gifford et al., 2017). Some bimodal users experience interference (Mussoi and Bentler, 2017; Goupell et al., 2018). A primary reason for the inconsistent fitting outcome is the lack of knowledge regarding the exact cues driving a bimodal benefit. This limitation seriously prohibits the development of an efficient frequency fitting scheme. The results obtained from the current study serve as control data for future bimodal studies with a plan to determine the frequency and time ranges responsible for bimodal benefit and interference in consonant recognition on an individual, subject-by-subject basis. Data sets from both studies will be used to train a neural network-based deep machine learning algorithm, which can cope up with the complexity of data that will be generated. One of the key components for the development of artificial intelligence-based algorithms is the availability and volume of high-quality data inputs. High-performing deep machine learning algorithms require high-quality data to learn which data set variables are most important for maximizing algorithm accuracy and minimizing errors (Vaerenberg et al., 2011; Wang, 2017; Wathour et al., 2020). Training the deep machine learning algorithm will be effective as the target and conflicting ranges are the individualized “right” answer for each consonant to the algorithm. This testing protocol is also possibly applied to vowel confusions to see a more holistic approach to this novel target and conflicting range-based fitting procedure for bimodal hearing in the future.

## Data Availability Statement

The original contributions presented in the study are included in the article/Supplementary Material, further inquiries can be directed to the corresponding author.

## Ethics Statement

The studies involving human participants were reviewed and approved by Texas Tech University Health Sciences Center. The patients/participants provided their written informed consent to participate in this study.

## Author Contributions

Y-SY conceived and designed the study, conducted the experiments, analyzed the data, and wrote the draft of the manuscript.

## Conflict of Interest

The author declares that the research was conducted in the absence of any commercial or financial relationships that could be construed as a potential conflict of interest.

## Publisher’s Note

All claims expressed in this article are solely those of the authors and do not necessarily represent those of their affiliated organizations, or those of the publisher, the editors and the reviewers. Any product that may be evaluated in this article, or claim that may be made by its manufacturer, is not guaranteed or endorsed by the publisher.
