# Supplementary figures and images for: Effect of the Target and Conflicting Frequency and Time Ranges on Consonant Enhancement in Normal-Hearing Listeners
Source: Front Psychol. 2021 Nov 15;12:733100. doi: 10.3389/fpsyg.2021.733100 (PMC8634346; doi:10.3389/fpsyg.2021.733100)

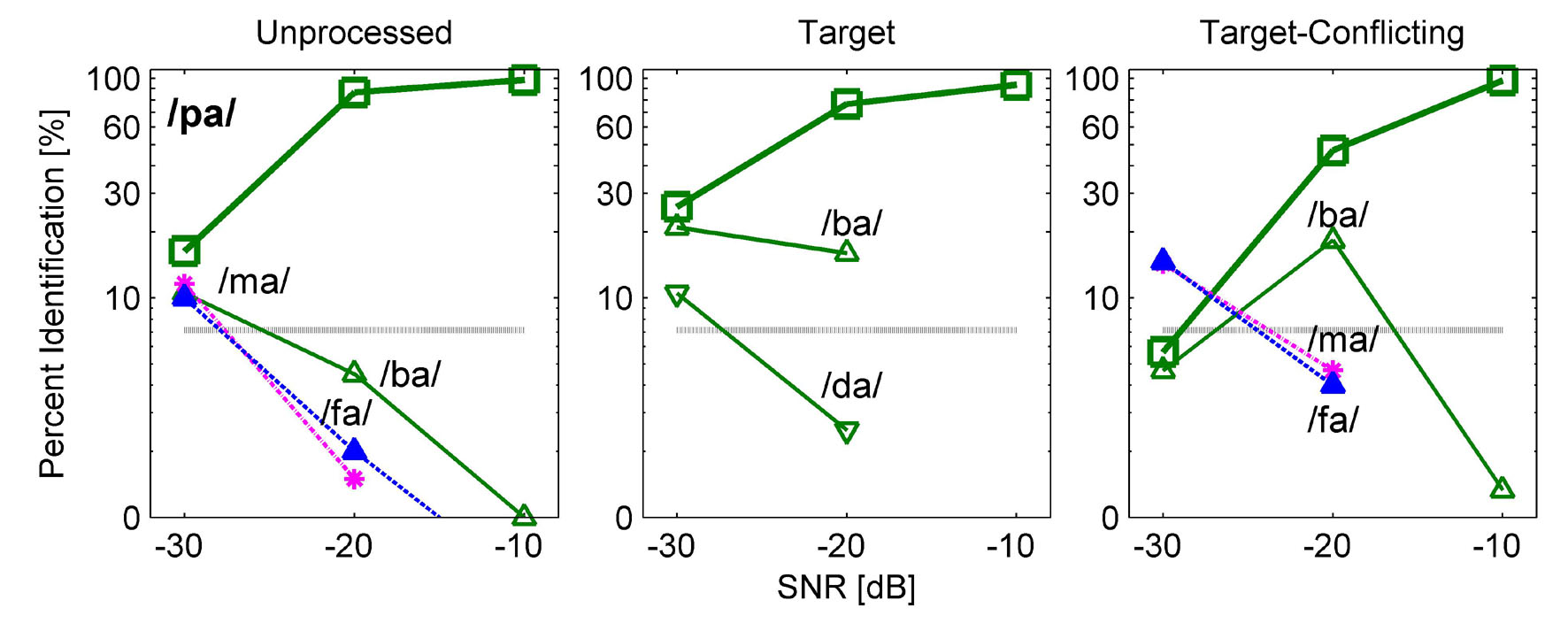

Supplement: Supplementary file 1 [file Image_1.JPEG]

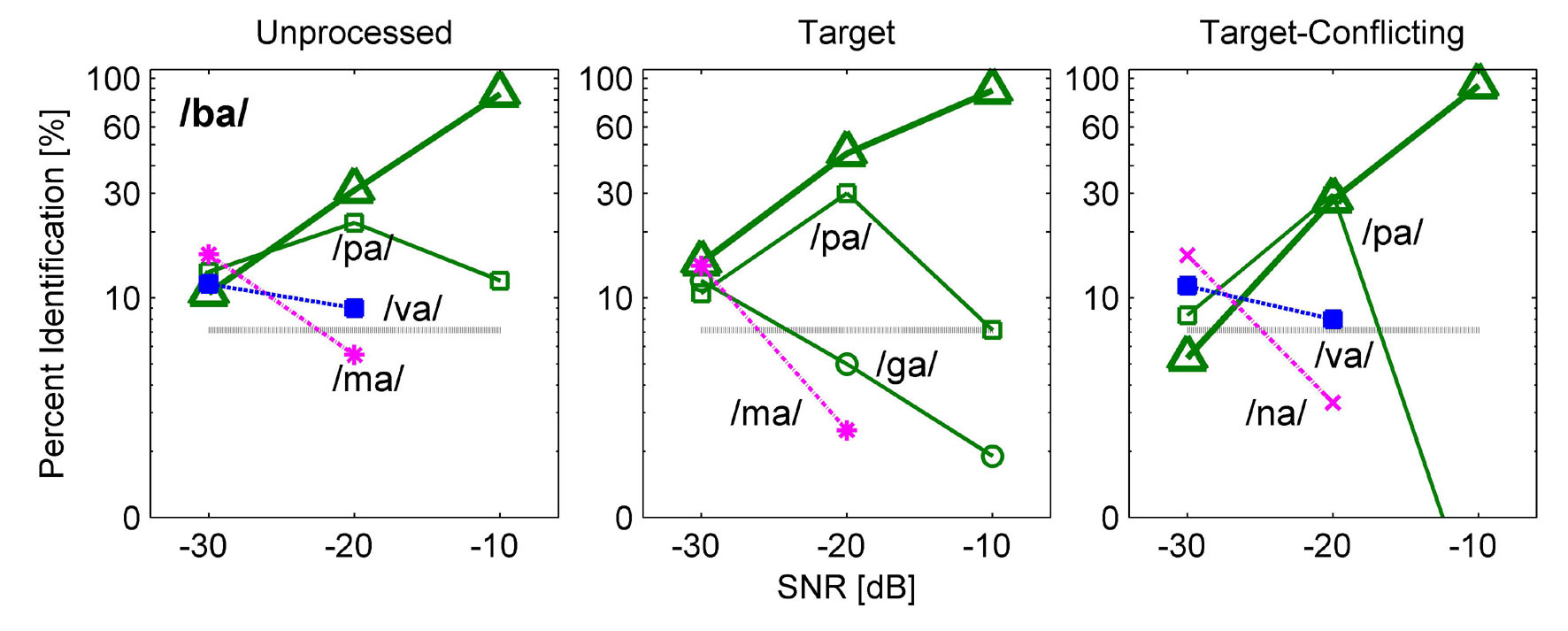

Supplement: Supplementary file 2 [file Image_2.JPEG]

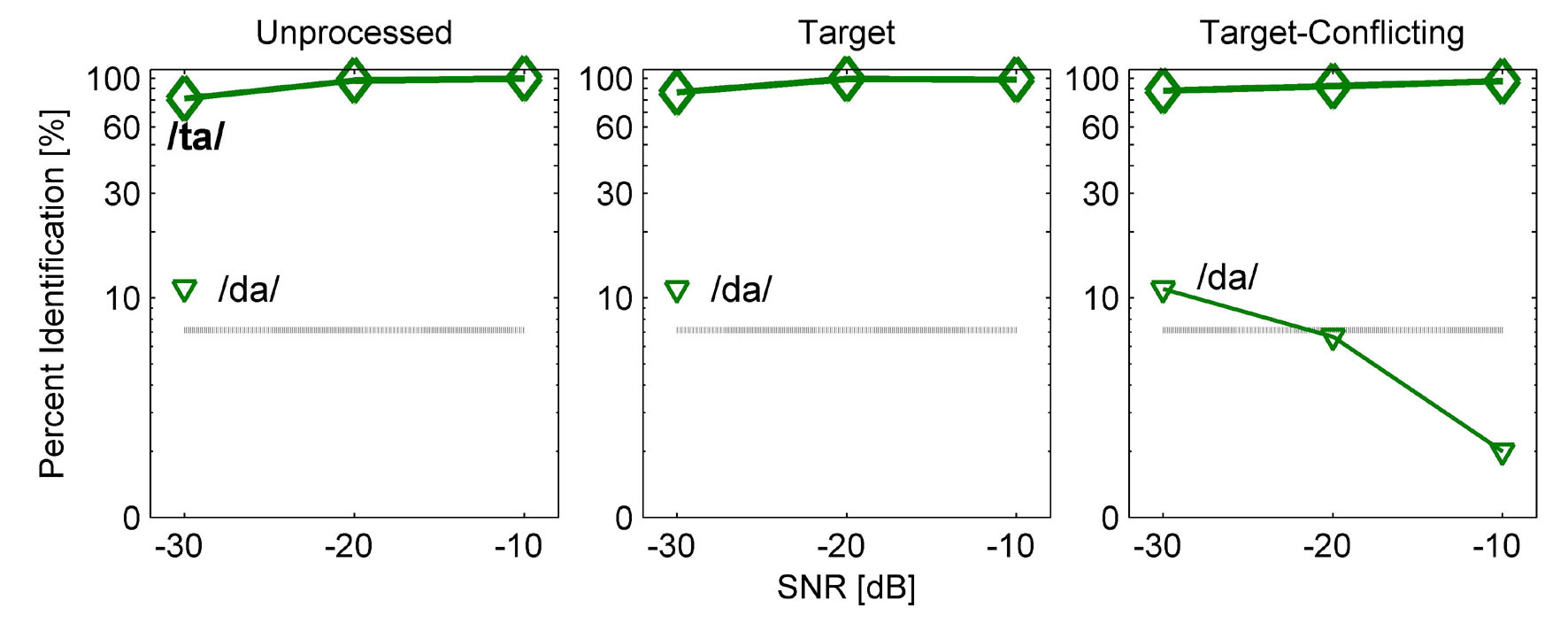

Supplement: Supplementary file 3 [file Image_3.JPEG]

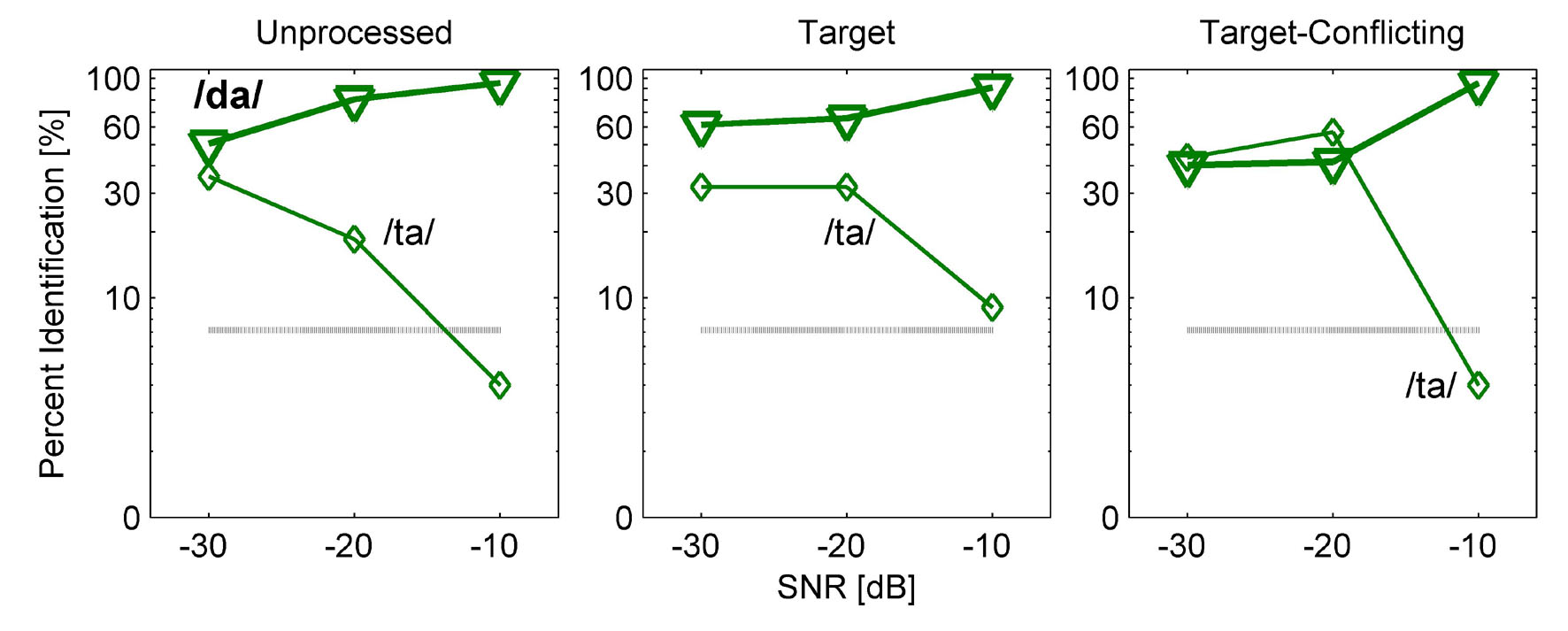

Supplement: Supplementary file 4 [file Image_4.JPEG]

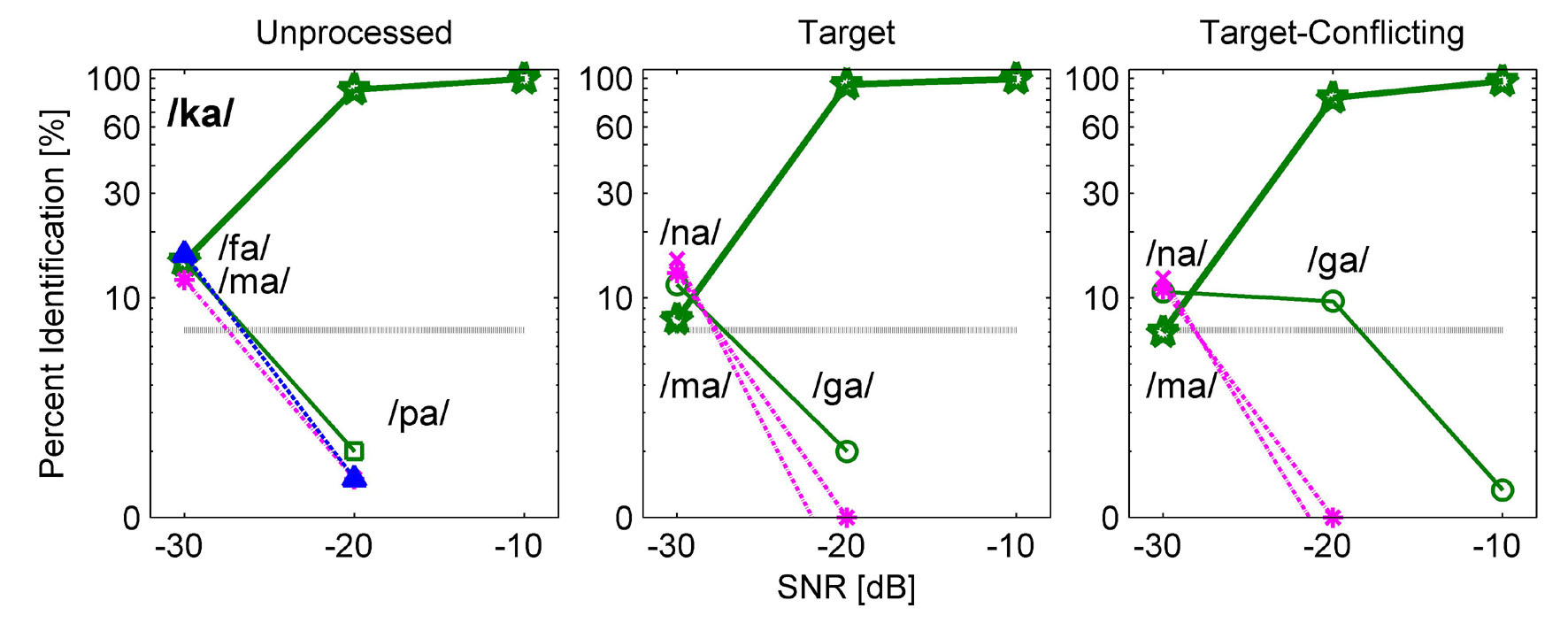

Supplement: Supplementary file 5 [file Image_5.JPEG]

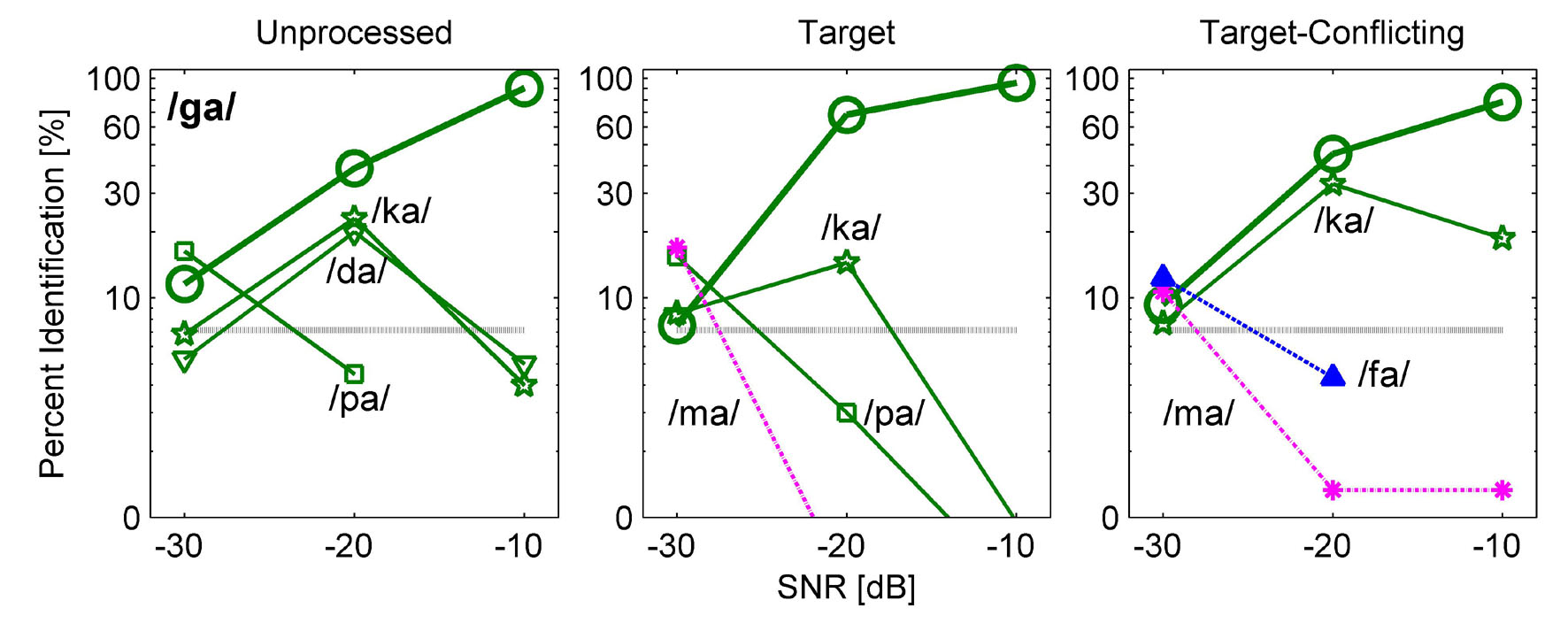

Supplement: Supplementary file 6 [file Image_6.JPEG]

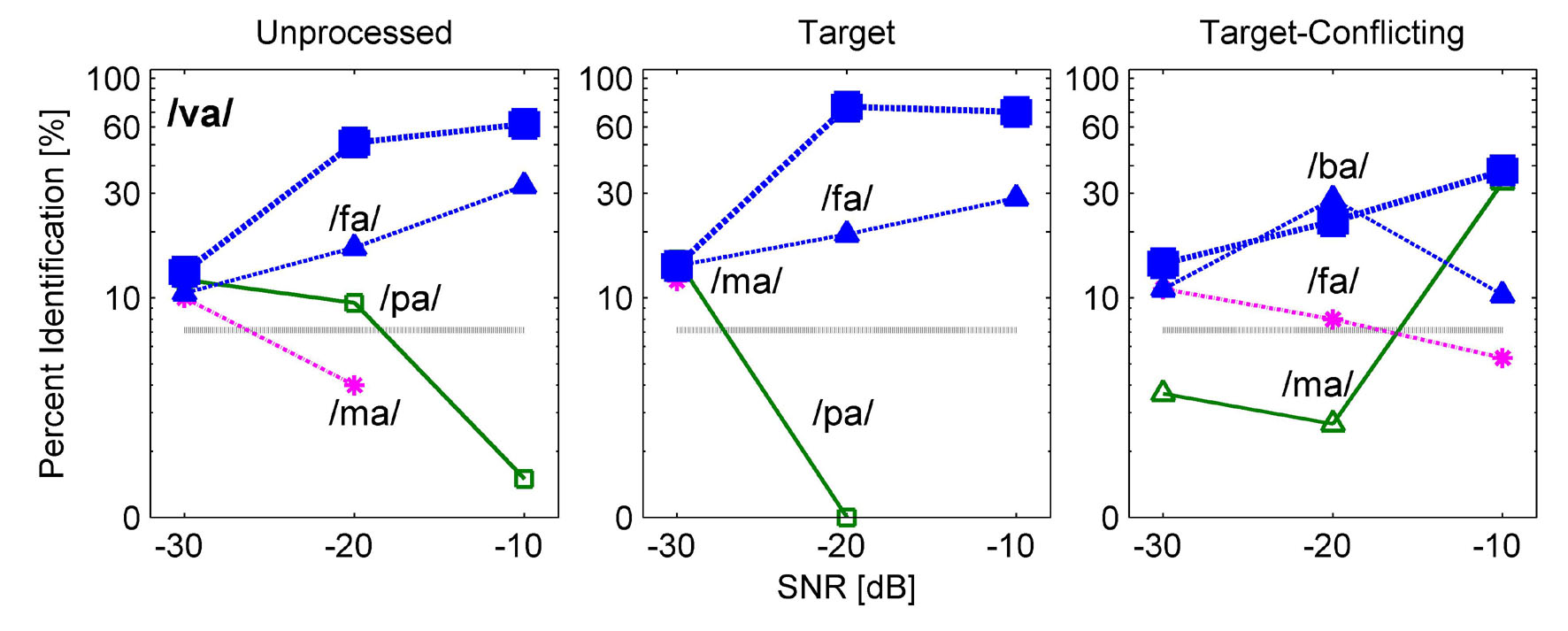

Supplement: Supplementary file 7 [file Image_7.JPEG]

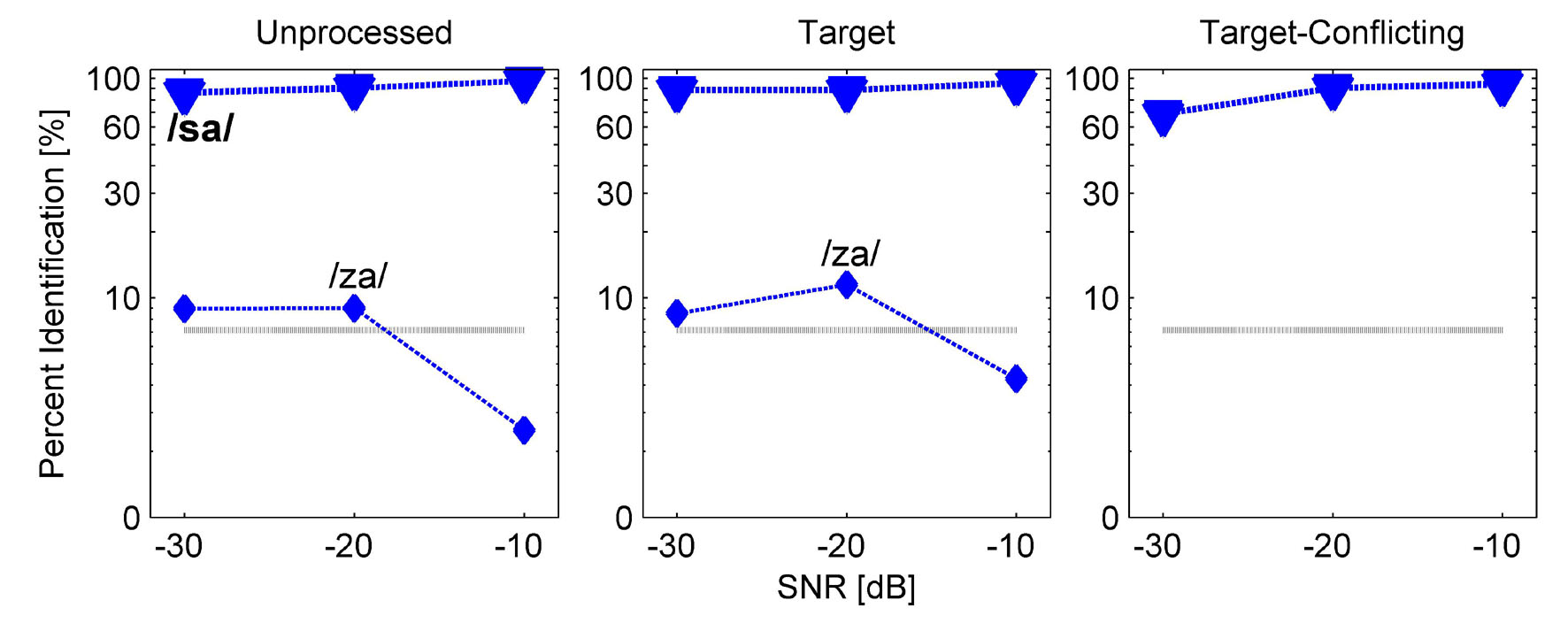

Supplement: Supplementary file 8 [file Image_8.JPEG]

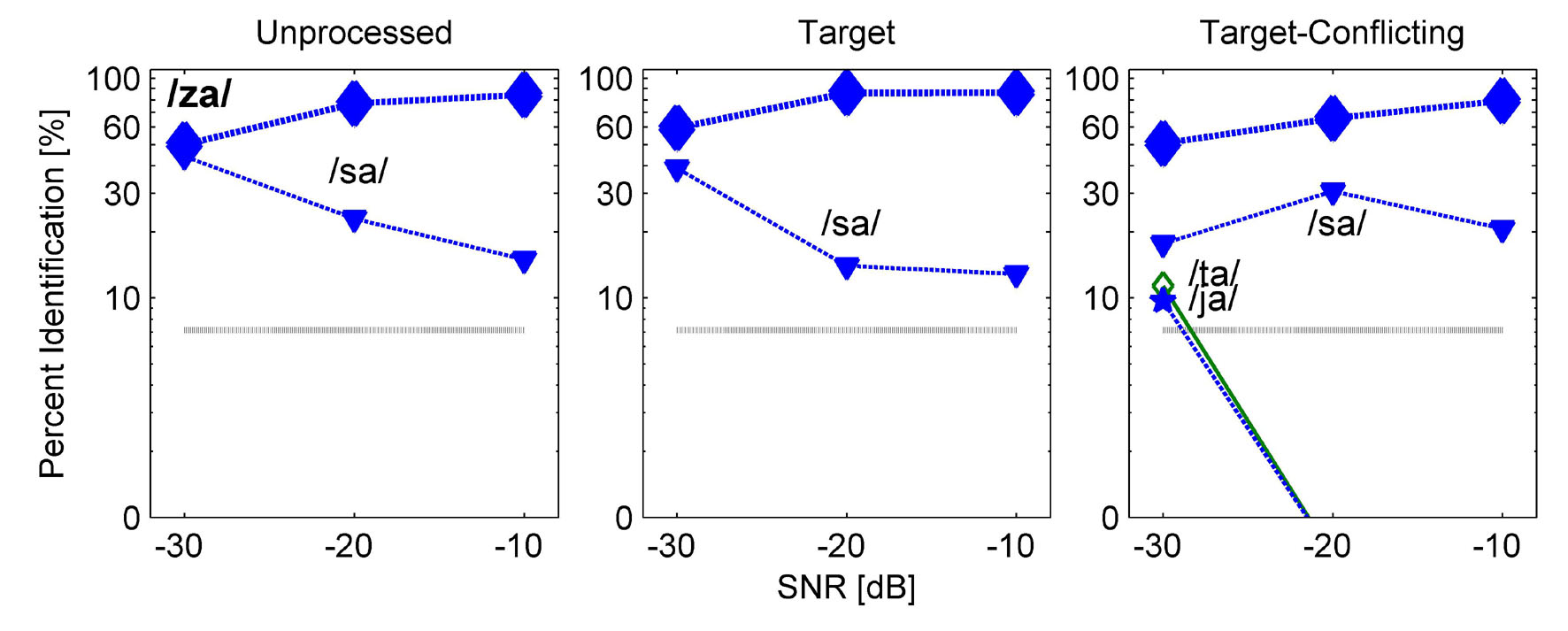

Supplement: Supplementary file 9 [file Image_9.JPEG]

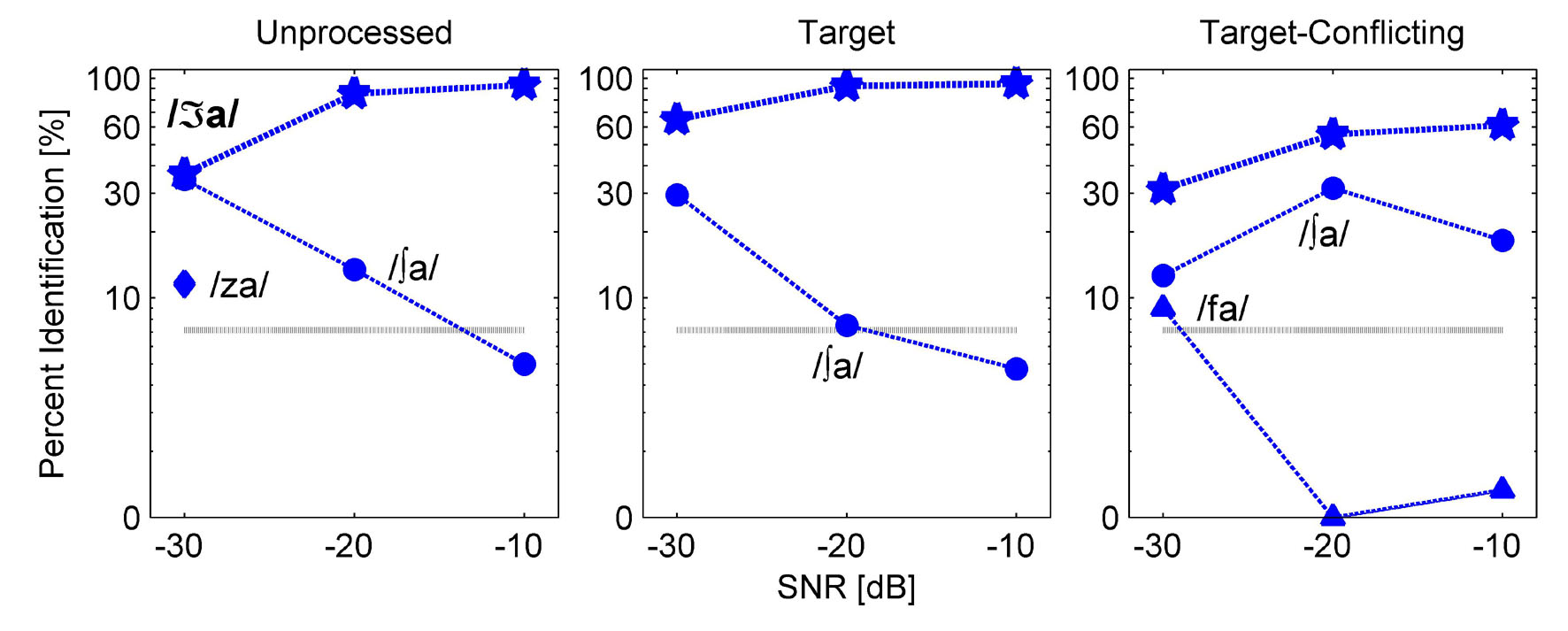

Supplement: Supplementary file 10 [file Image_10.JPEG]
